# Global Prevalence of Asthma in Adults with Atopic Dermatitis and the Atopic Dermatitis–Asthma Association

**DOI:** 10.3390/ph19081149

**Published:** 2026-07-24

**Authors:** Haojie Xu, Yichen Liu, Jiachen Tu, Mengmeng Liu, Libo Zhao

**Affiliations:** 1 Department of Pharmacy, Peking University Third Hospital, Beijing 100191, China; x17852205820@163.com (H.X.); blue189183417@163.com (Y.L.); tjc771816869@163.com (J.T.); 2511210484@bjmu.edu.cn (M.L.); 2 Department of Pharmacy Administration and Clinical Pharmacy, Peking University, Beijing 100191, China; 3 Research Group of Jian Gong on Pharmacoepidemiology and Clinical Drug Evaluation, School of Clinical Pharmacy, Shenyang Pharmaceutical University, Shenyang 110016, China

**Keywords:** asthma, atopic dermatitis, prevalence, comorbidity, pharmacotherapy

## Abstract

**Background:** Atopic dermatitis (AD) and asthma are common type 2 inflammatory diseases linked by the atopic march. In adults with AD, the prevalence of comorbid asthma and the strength of the AD–asthma association remain uncertain, with marked heterogeneity across studies. Moreover, the role of modern systemic therapies in this comorbidity is increasingly debated. **Objective:** To estimate the global pooled prevalence of asthma in adults with AD, quantify the AD–asthma association, and discuss the potential impact of targeted therapies (dupilumab, abrocitinib, omalizumab) on asthma comorbidity. **Methods:** Systematic review and random-effects meta-analysis (ID CRD420261304804) of observational studies from PubMed, EMBASE, and Cochrane Library (inception to 19 January 2026). Pooled prevalence and odds ratios (ORs) were calculated, with subgroup analyses by region, ethnicity, and disease definition. **Results:** Seventy-five studies were included. The global pooled prevalence of asthma in adults with AD was 25.9% (95% CI 22.1–30.0%; I^2^ = 99.9%). AD was significantly associated with asthma (OR 3.64, 95% CI 2.89–4.57; I^2^ = 98.6%). Prevalence varied from 9.3% in Asia to 63.2% in South America. Although dupilumab and other targeted agents are effective in both diseases, isolated reports of new-onset asthma after treatment were identified; these cases more likely reflect the natural progression of AD or unmasking of pre-existing asthma than a direct adverse drug reaction. **Conclusions:** Asthma is highly prevalent in adults with AD and is strongly associated with AD. Geographic and ethnic disparities call for stratified screening. Clinicians should be aware that new-onset respiratory symptoms during targeted therapy may represent AD-related comorbidity rather than drug-induced asthma.

## 1. Introduction

Atopic dermatitis (AD) is a common chronic inflammatory skin disease affecting approximately 3% to 10% of adults globally, while asthma affects over 300 million individuals worldwide [1,2,3]. The frequent co-occurrence of these two conditions is well-established and traditionally attributed to the “atopic march,” wherein infantile AD often precedes the development of asthma and allergic rhinitis [4,5,6]. Genetic studies have confirmed their shared basis, including filaggrin gene mutations and the IL-4/KIF3A loci [7]. Immunologically, type 2 inflammation mediated by interleukin (IL)-4 and IL-13 drives IgE production and eosinophil activation, playing a central role in both diseases [8,9].

Current treatment options for moderate-to-severe AD include biologics (e.g., the IL-4Rα antagonist dupilumab) [10,11] and oral JAK1 inhibitors (e.g., abrocitinib) [12]. Dupilumab has demonstrated efficacy in both AD [10] and asthma [13,14] in phase 3 clinical trials. For severe asthma, add-on biologics such as the anti-IgE agent omalizumab and the anti-IL-5 agent mepolizumab are available [15,16,17]. Given that AD and asthma share type 2 inflammatory pathways, targeted therapies against this pathway offer a theoretical rationale for concurrently addressing both conditions [5,18,19].

Although the association between AD and asthma is well recognized, previous systematic reviews have predominantly focused on pediatric populations [20,21] or have not specifically quantified asthma comorbidity in adult AD patients [22]. A prior meta-analysis by Ravnborg et al. reported a pooled asthma prevalence of 25.7% in mixed pediatric and adult populations, but evidence specifically in adults remains limited [23,24]. Unlike pediatric AD, which often resolves spontaneously before adolescence, adult AD tends to follow a chronic or relapsing course with distinct phenotypes, longer disease duration, and a higher burden of age-related comorbidities. Therapeutic strategies and treatment responses also differ between adults and children, with adults more frequently receiving systemic immunomodulators or biologics that may modify asthma comorbidity. Therefore, findings from pediatric or mixed populations cannot be directly extrapolated to adults, underscoring the need for this population-specific meta-analysis [25]. A systematic review and meta-analysis focused exclusively on adults with AD is warranted due to the wide variation in reported prevalence, the predominance of mixed-population studies in prior meta-analyses, the lack of systematic quantification of key moderators in adults, and the emergence of novel systemic therapies necessitating a contemporary synthesis. And adult AD patients reported asthma prevalence estimates vary widely across studies, ranging from 5% to over 50% [26,27], introducing uncertainty into clinical risk assessment and screening strategies [28,29]. Furthermore, comprehensive meta-analytic evidence on how factors such as ethnicity, geographic region, disease definition, and severity influence prevalence estimates remain lacking [9,30,31].

We conducted the largest systematic review and meta-analysis to date focusing exclusively on adults with AD to (1) determine the global pooled prevalence of comorbid asthma, (2) quantify the strength of the AD–asthma association, and (3) examine whether factors such as ethnicity, geographic region, and disease definition contribute to heterogeneity in prevalence estimates.

## 2. Methods

### 2.1. Literature Search

A study protocol was developed before the start of the study and registered online at PROSPERO (ID CRD420261304804). We followed the Preferred Reporting Items for Systematic Reviews and Meta-Analyses (PRISMA) guidelines. The medical databases PubMed, EMBASE, and Cochrane Library were searched using the search terms listed in Supplementary Table S1. The search covered all English-language articles from database inception until 19 January 2026. Additionally, reference lists of included studies and a prior systematic review on the same topic were manually screened to identify additional eligible studies.

All studies containing data on (1) the prevalence of asthma in adult patients (≥18 years) with atopic dermatitis or (2) the association between AD and asthma in adults were included. Exclusion criteria were: (1) case reports, case series, editorials, or conference abstracts; (2) animal studies or in vitro studies; (3) studies not published in English; and (4) studies with overlapping study populations (in which case the most comprehensive dataset was retained).

No restrictions were applied regarding specific age ranges, study types, or publication years.

### 2.2. Data Extraction

Two authors (H.X. and Y.L.) independently screened titles, abstracts, and full texts for eligibility. Disagreements were resolved by consultation with a third author (J.T.). Duplicates were removed, as were articles analyzing the same study population; when overlapping populations were identified, the article with the most comprehensive dataset was retained. Data extraction was performed using a standardized data extraction form (Supplementary Tables S2 and S3).

### 2.3. Quality Assessment

Study quality was assessed using the Newcastle-Ottawa Scale (NOS). For cohort and case–control studies, the original NOS (maximum 9 points) was used; for cross-sectional studies, an adapted version (maximum 10 points) was applied. Two authors (HX and YL) independently assessed quality, with disagreements resolved by JT. Studies were considered high quality if they scored ≥ 7 points, regardless of the NOS version. This threshold was used for subgroup analyses and sensitivity analyses. Clinical trials were not assessed with the NOS. Detailed quality assessment criteria are provided in Supplementary Table S4.

### 2.4. Statistical Analysis

We performed a proportion meta-analysis to obtain pooled prevalence estimates of asthma in adult patients with AD, and calculated odds ratios (ORs) with 95% confidence intervals (CIs) for the association between AD and asthma compared with reference individuals. All pooled estimates were derived using random-effects models according to the DerSimonian-Laird method, as substantial heterogeneity was anticipated across studies. Heterogeneity was assessed using Cochran’s Q test and the I^2^ statistic; an I^2^ > 50% and a Q-test *p* < 0.10 were considered indicative of significant heterogeneity.

### 2.5. Sensitivity Analysis

To evaluate the robustness of the pooled results, we conducted leave-one-out sensitivity analyses by sequentially excluding each study and recalculating the pooled estimates using random-effects models. We examined changes in pooled effect sizes, 95% CIs, and heterogeneity metrics (τ^2^ and I^2^). A study was considered influential if its exclusion resulted in a pooled effect estimate whose 95% CI no longer overlapped with that of the overall analysis, or if the I^2^ decreased by more than 5%.

### 2.6. Publication Bias and Graphical Presentation

Publication bias was assessed using funnel plots (Supplementary Figures S1 and S2), Egger’s regression test, and Begg’s rank correlation test. Forest plots were constructed to display individual study effect sizes, weights, and pooled estimates. All statistical analyses were performed using R version 4.5.1.

## 3. Results

**Figure 1:**
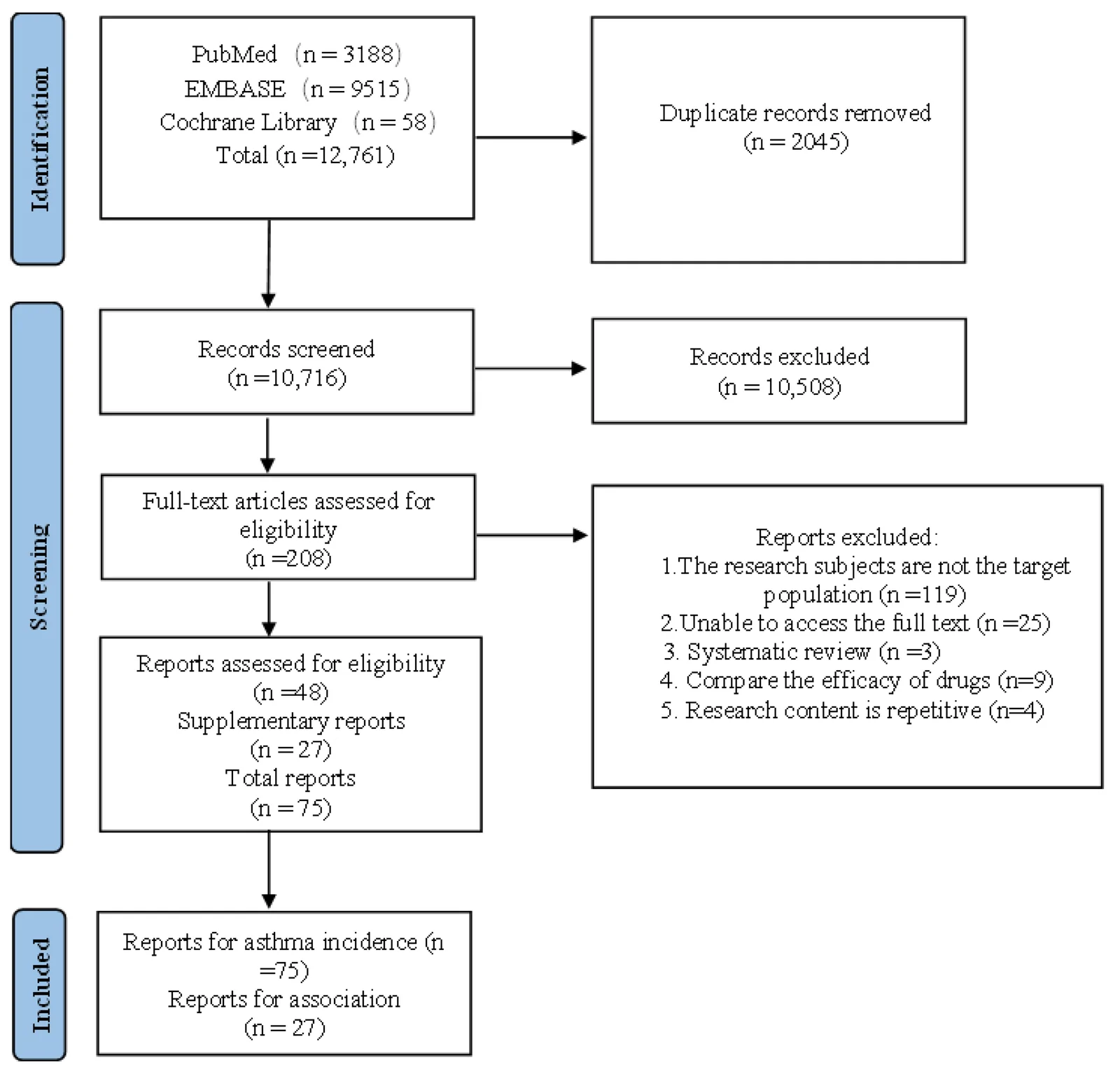
Preferred Reporting Items for Systematic Reviews and Meta-analysis (PRISMA) flow diagram of literature review and study selection.

A total of 12,761 articles were identified (PubMed, n = 3188; EMBASE, n = 9515; Cochrane Library, n = 58). After removing duplicates, 10,716 articles remained. Following title and abstract screening, 208 articles were reviewed in full text. Of these, 160 articles were excluded for reasons listed in the PRISMA flow diagram (Figure 1). Subsequently, 27 articles were supplemented based on previous studies, resulting in a total of 75 included studies. Among these, 75 studies were used to analyze the prevalence of asthma in adult patients with AD, and 25 studies were used to analyze the association between AD and asthma. The funnel plots for both meta-analyses were generally symmetric (Supplementary Figures S1 and S2). For the prevalence analysis, Egger’s test (*p* = 0.836) and Begg’s test (*p* = 0.2312) showed no significant publication bias. For the association analysis, Begg’s test (*p* = 0.65) showed no bias, while Egger’s test (*p* < 0.01) indicated potential bias, likely due to the substantial heterogeneity between studies. Overall, given the large dataset and the symmetric distribution of data across the assessment plots, no significant publication bias was evident. Leave-one-out sensitivity analyses were performed to assess the robustness of the pooled estimates (Supplementary Tables S5 and S6). For asthma prevalence, sequential exclusion of individual studies yielded pooled prevalence estimates that remained consistently close to the overall result, and all 95% confidence intervals overlapped with those of the full analysis. For the association between atopic dermatitis and asthma, the pooled odds ratios after excluding each study also remained within the confidence interval of the overall estimate, and no single study altered the direction or statistical significance of the association. Exclusion of any single study reduced I^2^ by less than 5%, confirming that no single study dominated the heterogeneity. These findings indicate that the meta-analysis results are robust to the influence of any individual study. Heterogeneity ranged from 63.5% to 99.9% across the proportion analyses and all subgroup analyze.

### 3.1. Prevalence of Asthma in Adults with Atopic Dermatitis

The pooled prevalence of asthma among 1,654,266 adults with AD was 25.9% (95% CI, 22.1–30.0%), with significant heterogeneity (I^2^ = 99.9%). Forest plot is in Supplementary File S2.

Geographic region and ethnicity emerged as the dominant sources of this heterogeneity. Prevalence estimates varied more than six-fold across regions, from a low of 9.3% (95% CI, 4.5–18.2%) in Asia to a high of 63.2% (95% CI, 24.0–90.3%) in South America, although the latter estimate was derived from only two studies and had wide confidence intervals. Intermediate estimates were observed in Europe (24.8%; 95% CI, 19.1–31.5%) and North America (37.3%; 95% CI, 25.6–50.8%) (Table 1, Supplementary Figure S3). This geographic pattern was mirrored in descriptive analyses by reported ethnic background: Asian populations had the lowest prevalence (11.1%; 95% CI, 4.9–23.3%), compared with predominantly White populations (26.3%; 95% CI, 21.1–32.2%) and mixed populations (33.2%; 95% CI, 22.5–46.0%). But these descriptive comparisons should be interpreted with caution, as the categories reflect broad population groupings rather than detailed ethnic classifications.

Disease definition also influenced estimates, albeit to a lesser extent. Studies relying on self-reported diagnoses yielded slightly higher prevalence than those using physician confirmation for both AD (25.3% vs. 22.5%) and asthma (26.1% vs. 23.5%), although confidence intervals overlapped considerably. In contrast, studies employing combined or ambiguous definitions produced markedly higher but imprecise estimates (AD: 69.2% [95% CI, 19.8–95.3%]; asthma: 37.6% [95% CI, 29.1–46.9%]), highlighting the importance of standardized diagnostic criteria. Beyond prevalence, we also quantified the strength of the AD–asthma association to contextualize the relative risk compared with individuals without AD.

### 3.2. Association Between Atopic Dermatitis and Asthma

**Figure 2:**
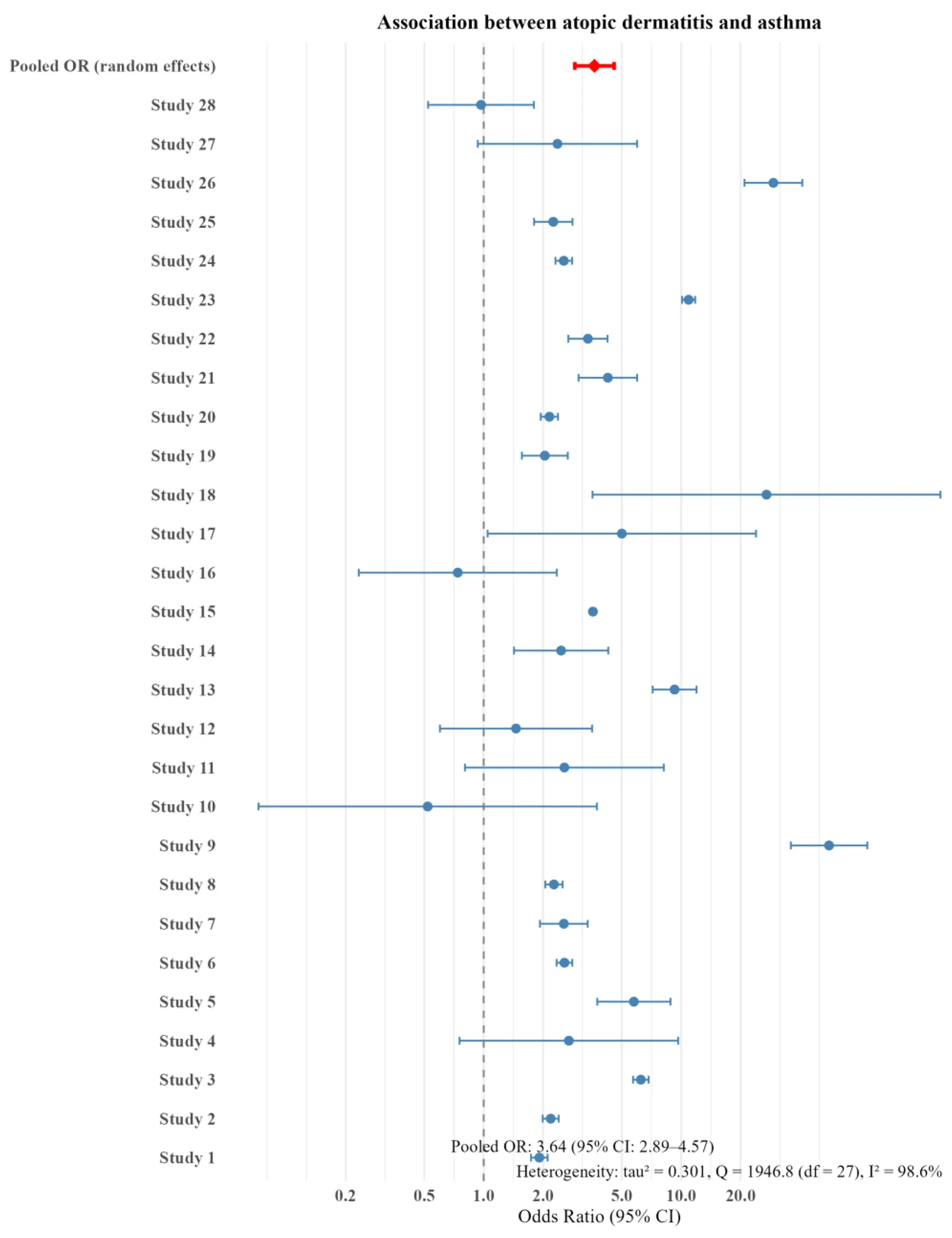
Forest plot of association between atopic dermatitis and asthma. The complete list of the 28 studies (Study 1 to Study 28) is provided in Supplementary Table S7.

Across 28 studies (from 25 articles) comprising 89,540 participants (35,322 with AD and 54,218 controls), AD was associated with a significantly increased odds of asthma. The pooled random-effects odds ratio was 3.64 (95% CI, 2.89–4.57; I^2^ = 98.6%; Figure 2), indicating a nearly 4-fold higher risk of asthma among adults with AD compared with those without.

The positive association was consistently observed across all predefined subgroups, underscoring its robustness. Stratification by geographic region yielded odds ratios ranging from 2.66 (95% CI, 2.11–3.35) in Asia to 5.32 (95% CI, 2.87–9.87) in North America, with Europe showing an intermediate estimate (OR, 3.33; 95% CI, 2.38–4.67) (Table 2, Supplementary Figure S4). Notably, studies from Asia demonstrated substantially lower heterogeneity (I^2^ = 63.5%) compared with other regions (all I^2^ > 98%), suggesting more consistent estimates in Asian populations.

The association remained significant irrespective of diagnostic criteria. Physician-diagnosed AD (OR, 3.27; 95% CI, 2.45–4.37) and self-reported AD (OR, 3.33; 95% CI, 2.05–5.41) yielded similar effect sizes, as did physician-diagnosed asthma (OR, 3.70; 95% CI, 2.80–4.88) and self-reported asthma (OR, 3.46; 95% CI, 1.66–7.22). By study design, both cross-sectional (OR, 3.49; 95% CI, 2.82–4.32) and cohort studies (OR, 2.84; 95% CI, 1.50–5.37) confirmed the positive association, although the latter showed wider confidence intervals.

Disease severity emerged as an important modifier of the association. In the 10 studies that explicitly reported on patients with moderate-to-severe AD, the pooled OR was 2.91 (95% CI, 2.37–3.57), with substantially reduced heterogeneity (I^2^ = 81.6%) compared to the overall analysis. This finding suggests that variability in AD severity across studies may account for a substantial portion of the observed heterogeneity.

The robustness of the primary finding was further supported by sensitivity analyses. The association persisted regardless of control type (pure control: OR, 3.99 [95% CI, 3.13–5.08]; general population: OR, 3.00 [95% CI, 1.59–5.67]) and study quality (NOS ≥ 7: OR, 3.69 [95% CI, 2.28–5.97]; NOS < 7: OR, 3.59 [95% CI, 2.79–4.60]). Funnel plot inspection and Begg’s test revealed no significant publication bias, although Egger’s test was suggestive of potential bias (*p* = 0.04), likely attributable to the substantial between-study heterogeneity rather than true publication bias.

## 4. Discussion

In this largest-to-date meta-analysis focusing exclusively on adults, the pooled prevalence of asthma in adult AD patients was 25.9%, corresponding to a 3.6-fold increased risk. This estimate is consistent with the 25.7% reported by Ravnborg et al. in a mixed pediatric and adult population [23], reinforcing the robustness of the association. However, our study extends prior knowledge by demonstrating marked geographic and ethnic variation, with prevalence ranging from 9.3% in Asia to 63.2% in South America, and providing more precise estimates across subgroups, enabled by a sample size more than double that of previous syntheses. These findings remained robust despite substantial interstudy heterogeneity.

The marked geographic variation, particularly the lower prevalence observed in Asian populations, may reflect underlying differences in genetic susceptibility and immune endotypes, including the Th17-skewed phenotype more frequently reported in Asian patients with AD. Meanwhile, the consistency between our adult-specific estimate and the mixed-population estimate reported by Ravnborg et al. does not diminish the value of population-specific investigation in adults, as the underlying disease mechanisms and clinical trajectories differ substantially between age groups [23]. Childhood-onset AD is frequently driven by the atopic march and may remit before adolescence in a substantial proportion of cases, whereas AD that persists into adulthood or presents with adult onset more commonly follows a chronic course characterized by distinct clinical phenotypes, longer disease duration, and a higher burden of comorbidities. The substantially larger sample size of the present study (over 1.6 million participants) enhances the precision of subgroup estimates, strengthening the evidence base for region-specific risk stratification. The robustness of our findings despite high interstudy heterogeneity further supports the validity of the observed associations, as the consistency of the direction and magnitude of effects across diverse study designs and populations argues against the results being driven by any single source of bias.

Although AD and asthma have traditionally been studied separately, they may share one or more common immune pathways [32,33]. The significant positive association observed in this study (OR = 3.64) further supports this notion. The shared immune dysfunction in AD and asthma, particularly type 2 immunity and elevated serum IgE levels, may explain their coexistence [34,35,36,37]. The predisposition to both AD and asthma is likely driven by shared genetic and/or environmental factors; genome-wide association studies (GWAS) have identified numerous shared genetic loci between AD and asthma, including variants in genes related to the epidermal barrier and T2 immune response [38,39]. In recent years, the IL-4 receptor α-antagonist dupilumab has demonstrated significant efficacy in clinical trials for both AD [10,11,40] and asthma [13,14,41] further confirming the close connection between these two diseases in terms of immune pathways and providing a theoretical basis for exploring monotherapy for both conditions in selected patients [42,43].

Although the difference in prevalence between studies including severe AD patients (26.08%) and those without clear severity specification (24.12%) did not reach statistical significance in our analysis, previous large cohort studies have demonstrated an increasing trend in asthma prevalence with greater AD severity [23,44]. For example, in the SOLO 1 and 2 trials of dupilumab, the prevalence of a history of asthma among patients with moderate-to-severe AD even reached 49.5% [18,45]. Several systemic therapies are available for moderate-to-severe AD, including the IL-4Rα antagonist dupilumab, the IL-13 inhibitor tralokinumab, oral JAK inhibitors (abrocitinib, upadacitinib), and conventional immunosuppressants such as cyclosporine and methotrexate. Among these, dupilumab has demonstrated efficacy in both AD and asthma, while omalizumab and mepolizumab are primarily used in asthma [15,16]. Importantly, the following discussion on targeted therapies and new-onset asthma represents hypothesis-generating observations based on isolated case reports, rather than definitive conclusions from our meta-analysis. However, during the literature search, we identified isolated case reports of new-onset asthma occurring in patients following treatment with some of these agents [12,46,47]. Based on current pharmacological evidence, a causal relationship lacks biological plausibility for dupilumab, and for JAK inhibitors, current evidence is limited to isolated case reports] and does not support a definitive causal link. Prospective pharmacovigilance studies are needed to clarify this question [12,48].

First, it should be clarified that atopic dermatitis itself increases the risk of asthma through the “atopic march”. Therefore, new-onset asthma is more likely to reflect the natural progression of AD rather than a direct adverse drug reaction. Especially in patients with uncontrolled or severe AD, systemic type 2 inflammation is elevated, and airway remodelling may have already started insidiously before skin symptoms become apparent [8,9]. Consequently, the “unmasking” of previously unrecognized asthma after skin improvement may also result from a shift in clinical attention.

Second, from a pharmacological mechanism perspective, dupilumab blocks the IL-4/IL-13 signalling pathway and would theoretically suppress rather than induce asthma. A causal relationship with new-onset asthma cases lacks biological plausibility. In contrast, JAK1 inhibitors such as abrocitinib have broader effects on multiple cytokine pathways. Whether they can induce specific airway inflammation through immune remodelling remains unclear and is currently supported only by isolated case reports [12].

Another possibility is that drug exposure alters the phenotype of asthma. For example, dupilumab treatment can cause a transient increase in peripheral blood eosinophil counts, but in most studies this has not been associated with worsening of clinical symptoms [34]. Therefore, in the absence of clear airway inflammation evidence or challenge test results, attributing new-onset asthma to these drugs should be done with great caution.

Geographic region significantly influenced asthma prevalence, with Asia showing the lowest prevalence (9.3%), while North America (37.3%) and South America (63.2%) had higher estimates. Descriptive analyses by reported ethnic background showed similar patterns, with Asian populations having the lowest prevalence (11.1%) compared with predominantly White (26.3%) and mixed populations (33.2%). These differences may reflect underlying genetic, environmental, and healthcare-related factors. Notably, Asian patients with AD often exhibit a Th17-skewed phenotype with less pronounced type 2 inflammation [49,50], which may attenuate the risk of comorbid asthma. In contrast, the high prevalence in South American populations—though based on limited data—may be influenced by high aeroallergen exposure, climatic conditions, and variable healthcare access [51]. These hypotheses warrant further investigation in well-designed cohort studies.

Disease definition influenced prevalence estimates [52]. Studies using self-reported definitions yielded slightly higher estimates than those using physician diagnosis [53], while combined definitions produced substantially higher estimates (AD: 69.19%; asthma: 37.57%) with wide confidence intervals. This suggests that single definitions may underestimate true prevalence, whereas combined definitions, despite potentially capturing more cases, risk overdiagnosis [54,55].

The significant positive association between AD and asthma (OR = 3.64) was consistently observed across all subgroups, indicating the robustness of this relationship [54]. Notably, studies from Asia demonstrated considerably lower heterogeneity (I^2^ = 63.5%) compared with other regions (all I^2^ > 98%), suggesting more consistent findings in Asian populations. This may be attributed to the relatively smaller number of studies and more homogeneous genetic background in this region [56,57]. Furthermore, subgroup analyses stratified by AD severity also showed markedly reduced heterogeneity (I^2^ = 81.6%), indicating that disease severity is an important factor influencing both the association and heterogeneity [58].

Comorbidity significantly increases the clinical burden of AD patients, with higher infection risk and impaired quality of life. Thus, routine respiratory assessment in AD patients, and conversely skin examination in asthmatic patients, is warranted to avoid underdiagnosis. The shared pathogenesis of AD and asthma also offers opportunities for dual-effective therapies, such as dupilumab, which has demonstrated efficacy in both conditions [59]. IL-6 is a key factor in comorbidity, and these findings support further study of their shared pathogenesis to improve combined treatment [60].

## 5. Limitations

First, definitions of AD and asthma varied across studies, which may introduce misclassification bias. Second, inconsistent severity reporting precluded dose–response analysis. Third, substantial heterogeneity persisted despite subgroup analyses, suggesting unidentified sources. Fourth, most studies originated from high-income countries, limiting generalizability to other regions. Fifth, while funnel plots showed no publication bias for prevalence, Egger’s test for association was suggestive of potential bias, warranting caution. Finally, data on ethnic background were limited, with only one study reporting Black populations. Therefore, our subgroup comparisons are primarily geographic rather than ethnic, and the findings should be interpreted with caution.

## 6. Conclusions

This meta-analysis of 75 studies comprising over 1.6 million adults with AD demonstrates that the global pooled prevalence of asthma in adult AD patients is 25.9%, corresponding to a 3.6-fold increased risk compared with individuals without AD. Our study provides the first comprehensive prevalence estimates specifically in adult AD populations stratified by geographic region, revealing marked regional variation from 9.3% in Asia to 63.2% in South America. These findings suggest that Asian populations may have a lower comorbidity burden, potentially attributable to distinct immunological phenotypes. The results reaffirm the strong AD–asthma association in adults, consistent with the atopic march paradigm, and highlight the necessity of routine respiratory assessment in AD patients. Future investigations should adopt standardized diagnostic criteria to minimize heterogeneity, include underrepresented regions with larger sample sizes, and prospectively evaluate the impact of modern systemic therapies on asthma comorbidity trajectories in adult AD patients.

## Figures and Tables

**Table 1:** Subgroup analysis of asthma prevalence in adults with atopic dermatitis.

Variable	Subgroup	Study	Total	Events	Prevalence (%)	95% CI	I^2^ (%)	Q	τ^2^
Ethnicity	White	44	1,630,895	357,209	26.25	21.08–32.17	99.95	83,343.08	0.87
	Asian	9	7915	1256	11.07	4.85–23.31	98.93	747.02	1.75
	Mixed	8	4470	1236	33.21	22.51–45.98	97.46	275.19	0.53
Design	Cohort study	31	571,230	52,662	21.73	16.15–28.58	99.64	8431.55	0.96
	Cross-sectional study	21	1,060,887	304,437	23.17	19.39–27.44	99.62	5307.94	0.25
	Case–control study	10	11,272	2620	32.76	24.27–42.55	98.09	472.26	0.38
AD-definition	Doctor’s diagnosis	51	1,632,615	356,734	22.45	18.12–27.48	99.94	83,752.23	0.88
	Self-report	8	6861	1511	25.32	18.79–33.20	96.15	182.01	0.24
	Combined definition *	2	1003	508	69.19	19.79–95.34	98.92	92.86	2.51
Asthma-definition	Doctor’s diagnosis	52	1,634,978	357,335	23.45	19.02–28.55	99.94	83,949.97	0.87
	Self-report	8	4682	1071	26.07	18.02–36.13	96.09	179.15	0.39
	Combined definition *	2	3729	1313	37.57	29.08–46.90	95.73	23.42	0.07
NOS-score	High (≥7)	24	47,668	8793	25.63	20.38–31.69	99.24	3030.84	0.51
	Low (<7)	38	1,595,721	350,926	23.33	18.07–29.57	99.95	81,081.83	0.92
Region	Europe	32	1,626,489	355,843	24.76	19.07–31.48	99.96	82,731.41	0.87
	Asia	13	8315	1270	9.3	4.53–18.16	98.51	805.22	1.81
	North America	7	6631	1823	37.33	25.60–50.76	98.61	431.93	0.52
	Africa *	2	262	86	28.75	9.31–61.34	95.24	20.99	0.93
	Global	5	1370	487	33.05	21.93–46.45	91.45	46.8	0.32
	South America *	2	280	189	63.19	24.04–90.30	97.38	38.23	1.45
	Unclear	26	1,078,434	307,276	22.94	19.16–27.21	99.67	7463.18	0.3
Severe AD	Yes	33	558,035	51,209	26.08	19.40–34.08	99.58	7596.69	1.16
	No *	3	6920	1234	24.12	14.56–37.21	97.44	78.05	0.29

Note: * Subgroups with fewer than three studies; estimates should be interpreted with caution. Studies were grouped by reported ethnic background where available (White, Asian, Mixed). However, these categories reflect broad population groupings rather than detailed ethnic classifications, as the original studies did not provide sufficient granularity for ethnicity-level analyses. Subgroup analyses by geographic region are presented separately as the primary basis for regional comparisons.

**Table 2:** Subgroup analysis of association between atopic dermatitis and asthma.

Variable	Subgroup	Study	Total	Events	OR	95% CI	I^2^ (%)	τ^2^	Q
Design	Cohort	11	15,096	3597	2.836	1.50–5.37	99.09	0.97	1103.31
	Cross-sectional	15	74,181	13,033	3.489	2.82–4.32	97.67	0.14	599.52
	Case–control *	2	263	194	7.419	0.14–395.94	99.08	8.16	109.22
AD-definition	Physician-diagnosed	19	79,638	14,226	3.27	2.45–4.37	98.73	0.32	1416.86
	Self-reported	7	6808	1471	3.328	2.05–5.41	97.34	0.36	225.31
Asthma-definition	Physician-diagnosed	21	82,463	14,957	3.696	2.80–4.88	98.80	0.33	1669.96
	Self-reported	6	4167	901	3.461	1.66–7.22	97.52	0.73	201.71
Control-type	Pure control	20	73,884	13,886	3.985	3.13–5.08	97.94	0.22	922.96
	General population	8	15,656	2938	2.998	1.59–5.67	99.25	0.78	938.09
NOS-Score	High (≥7)	13	24,931	5004	3.693	2.28–5.97	99.20	0.73	1506.60
	Low (<7)	15	64,609	11,820	3.586	2.79–4.60	96.82	0.15	439.92
Region	Europe	14	75,947	14,054	3.332	2.38–4.67	99.07	0.33	1402.56
	Asia	8	7471	1206	2.661	2.11–3.35	63.53	0.04	19.20
	North America	4	5860	1478	5.322	2.87–9.87	98.51	0.38	201.13
	Africa *	2	262	86	8.589	0.76–97.12	98.23	3.01	56.62
Severe AD	No/Unclear	18	78,598	148,48	3.963	2.93–5.37	99.04	0.36	1775.87
	Yes	10	10,942	1976	2.909	2.37–3.57	81.58	0.06	48.87

Note: * Subgroups with fewer than three studies; estimates should be interpreted with caution. Studies were grouped by reported ethnic background where available (White, Asian, Mixed). However, these categories reflect broad population groupings rather than detailed ethnic classifications, as the original studies did not provide sufficient granularity for ethnicity-level analyses. Subgroup analyses by geographic region are presented separately as the primary basis for regional comparisons.

## Data Availability

The original contributions presented in this study are included in the article/supplementary material. Further inquiries can be directed to the corresponding author.

## Supplementary Materials

The following supporting information can be downloaded at: https://www.mdpi.com/article/10.3390/ph19081149/s1, Table S1: Literature search strategies; Table S2: Literature characteristics table of asthma prevalence; Table S3: Table of Literature characteristics on the association between asthma prevalence and atopic dermatitis; Table S4: NOS score of literature; Table S5: Sensitivity analysis of asthma prevalence; Table S6. Sensitivity analysis of AD -asthma association; Table S7. List of studies included in the meta-analysis (corresponding to Study 1–28 in Figure 2); Figure S1: Funnel plot of asthma prevalence. Figure S2: Funnel plot of association between asthma and atopic dermatitis; Figure S3: Subgroup analysis of asthma prevalence; Figure S4: Subgroup analysis of AD-asthma association; File S2: Prevalence forest plot.
