# Supplementary material for: Global Prevalence of Asthma in Adults with Atopic Dermatitis and the Atopic Dermatitis–Asthma Association
Source: Pharmaceuticals (Basel). 2026 Jul 24;19(8):1149. doi: 10.3390/ph19081149 (PMC13516457; doi:10.3390/ph19081149)

Prevalence of asthma in adults with atopic dermatitis

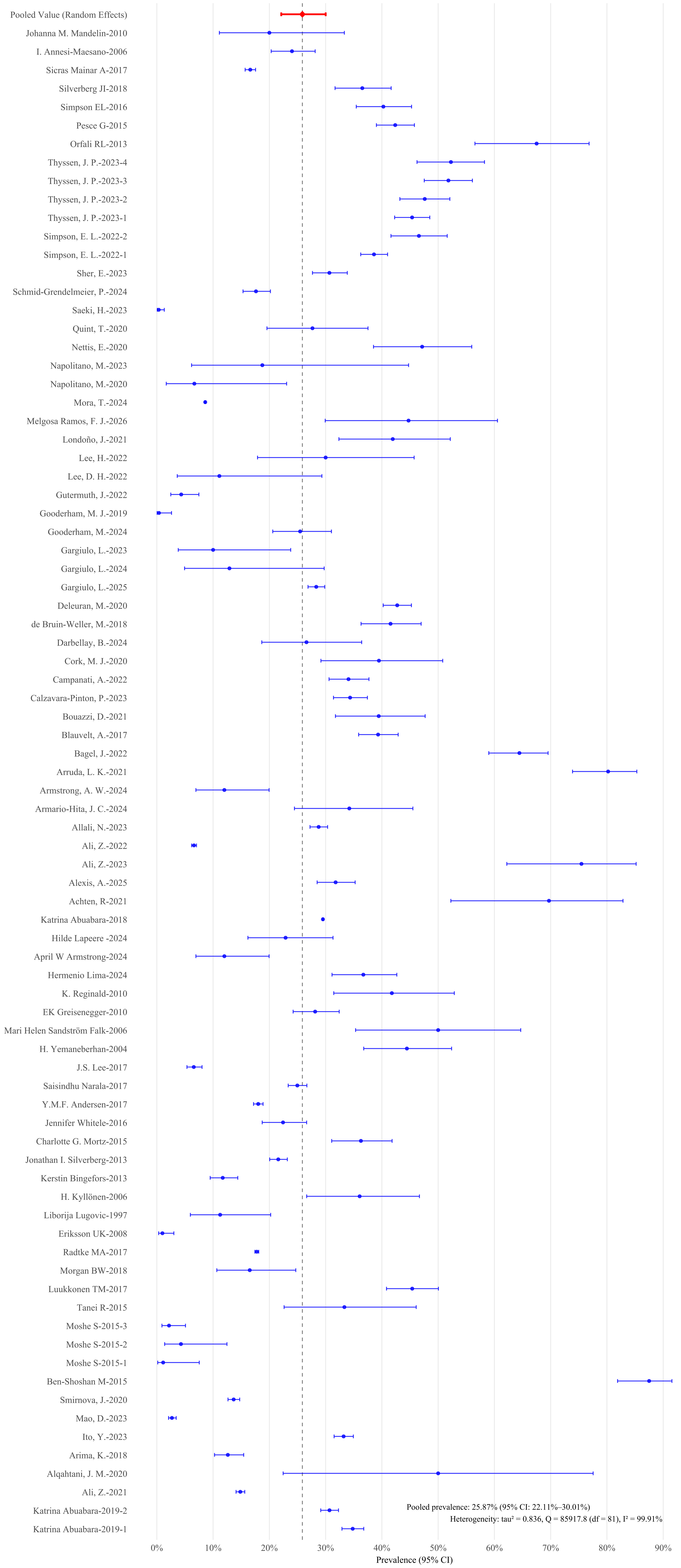

Supplement: Supplementary file 1 [file pharmaceuticals-19-01149-s001.zip › File S2 Prevalence forest plot.pdf]
