# Supplementary material for: Global Prevalence of Asthma in Adults with Atopic Dermatitis and the Atopic Dermatitis–Asthma Association
Source: Pharmaceuticals (Basel). 2026 Jul 24;19(8):1149. doi: 10.3390/ph19081149 (PMC13516457; doi:10.3390/ph19081149)
Supplement: Supplementary file 1 [file pharmaceuticals-19-01149-s001.zip › SA1.pdf]

Table S1. Literature search strategies.

Database	Search strategy
EMBASE	('adults'/exp OR 'adults' OR 'grown-ups'/exp OR 'grown-ups' OR 'grownup'/exp OR 'grownup' OR 'grownups'/exp OR 'grownups' OR 'adult'/exp OR 'adult') AND ('atopic dermatitis'/exp OR 'atopic dermatitis' OR 'dermatitis atopic'/exp OR 'dermatitis atopic' OR 'eczema'/exp OR 'eczema' OR 'childhood eczema'/exp OR 'childhood eczema' OR 'infantile eczema'/exp OR 'infantile eczema' OR 'neurodermatitis'/exp OR 'neurodermatitis' OR 'besnier prurigo' OR ('besnier' AND ('prurigo'/exp OR 'prurigo'))) AND ('asthma'/exp OR 'asthma' OR ('allergic' AND ('asthma'/exp OR 'asthma')) OR ('non-allergic' AND ('asthma'/exp OR 'asthma')) OR 'wheezing'/exp OR 'wheezing' OR ('reactive' AND ('airway'/exp OR 'airway')))
PUBMED	("dermatitis, atopic"[MeSH Terms] OR "atopic dermatitis"[All Fields] OR "dermatitis atopic"[All Fields] OR "Eczema"[MeSH Terms] OR "Eczema"[All Fields] OR "childhood eczema"[All Fields] OR "infantile eczema"[All Fields] OR "Neurodermatitis"[All Fields] OR "Neurodermatitis"[MeSH Terms] OR "Besnier's prurigo"[All Fields] OR ("Besnier's"[All Fields] AND "prurigo"[All Fields])) AND ("asthma"[MeSH Terms] OR "asthma"[All Fields] OR "allergic asthma"[All Fields] OR "non-allergic asthma"[All Fields] OR "wheezing"[All Fields] OR ("reactive"[All Fields] AND "airway"[All Fields])) AND ("adult"[MeSH Terms] OR "adult"[All Fields] OR "adults"[All Fields] OR "adult s"[All Fields])
EMBASE	#1 MeSH descriptor: [Dermatitis, Atopic] explode all trees<br>#2 (Atopic Dermatitis): ti,ab,kw<br>#3 (Eczema, Atopic): ti,ab,kw<br>#4 (Atopic Eczema): ti,ab,kw<br>#5 (Neurodermatitis, Atopic): ti,ab,kw<br>#6 (Atopic Neurodermatitis): ti,ab,kw<br>#7 (Eczema, Infantile): ti,ab,kw<br>#8 (Infantile Eczema): ti,ab,kw<br>#9 #1or#2or#3or#4or#5or#6or#7or#8<br>#10 MeSH descriptor: [Asthma] explode all trees<br>#11 (Asthmas): ti,ab,kw<br>#12 (Asthma, Bronchial): ti,ab,kw<br>#13 (Bronchial Asthma): ti,ab,kw<br>#14 MeSH descriptor: [Adult] explode all trees<br>#15 (Adults): ti,ab,kw<br>#16 #10or#11or#12or#13<br>#17 #14or#15#18 #9and#16and#17

Table S2. Literature characteristics table of asthma prevalence

Author+Year	Publication Year	Ethnity	Design	AD-Definition	Asthma-Definition	Total number in AD	Total male number in AD	Total asthma number in AD	NOS	Rate (%)	Region	Severity
Katrina Abuabara-2019-1	2019	White	Cohort study	Diagnosed	Diagnosed	2318	1046	807	7	34.8145	Europe	Unclear
Katrina Abuabara-2019-2	2019	White	Cohort study	Diagnosed	Diagnosed	3275	1463	1005	7	30.6870 2	Europe	Unclear
Ali, Z.-2021	2021	White	Cross-sectional study	Diagnosed	Diagnosed	8095	5002	1201	6	14.8363 2	Europe	Unclear
Alqahtani, J. M.-2020	2020	White	Cross-sectional study	Self-report	Self-report	10		5	6	50	Asia	Unclear
Arima, K.-2018	2018	Asian	Cross-sectional study	Diagnosed	Diagnosed	634	302	80	9	12.6183	Asia	Yes
Ito, Y.-2023	2023	Asian	Cross-sectional study	Diagnosed+ Self-report	Diagnosed+ Self-report	2910		966	6	33.1958 8	Asia	Yes
Mao, D.-2023	2023	Asian	Cross-sectional study	Diagnosed	Diagnosed	2202	872	59	10	2.67938 2	Asia	Yes
Smirnova, J.-2020	2020	White	Cross-sectional study	Diagnosed	Diagnosed	4175	1811	570	8	13.6526 9	Europe	Yes
Ben-Shoshan M-2015	2015	White	Case-control	Diagnosed+ Self-report	Diagnosed	184		161	7	87.5	North America	Unclear
Moshe S-2015-1	2015	White	Cohort study	Diagnosed	Diagnosed	89		1	6	1.12359 6	Asia	Unclear
Moshe S-2015-2	2015	White	Cohort study	Diagnosed	Diagnosed	70		3	6	4.28571 4	Asia	Unclear
Moshe S-2015-3	2015	White	Cohort study	Diagnosed	Diagnosed	231		5	6	2.16450 2	Asia	Unclear
Tanei R-2015	2015	Asian	Case-control	Diagnosed	Diagnosed	60	41	20	6	33.3333 3	Asia	Yes
Luukkonen TM-2017	2017	White	Cohort study	Diagnosed	Diagnosed	445		202	8	45.3932 6	Europe	Unclear
Morgan BW-2018	2018	Black	Cross-sectional study	Diagnosed	Diagnosed	109		18	7	16.5137 6	Africa	Unclear
Radtke MA-2017	2017	White	Cross-sectional study	Diagnosed	Diagnosed	48140		8553	6	17.7669 3	Europe	Unclear

Author+Year	Publication Year	Ethnity	Design	AD-Definition	Asthma-Definition	Total number in AD	Total male number in AD	Total asthma number in AD	NOS	Rate (%)	Region	Severity
Eriksson UK-2008	2008	White	Cohort study	Self-report	Self-report	303		3	9	0.990099	Europe	Unclear
Liborija Lugovic-1997	1997	White	Cohort study	Diagnosed	Diagnosed	80		9	6	11.25	Europe	Yes
H. Kyllönen-2006	2006	White	Cross-sectional study	Diagnosed	Diagnosed	86	29	31	5	36.04651	Europe	Yes
Kerstin Bingefors-2013	2013	White	Cross-sectional study	Self-report	Self-report	666	240	78	7	11.71171	Europe	Unclear
Jonathan I. Silverberg-2013	2013	Mixed	Cross-sectional study	Self-report	Diagnosed	2641	1044	570	7	21.58273	North America	Unclear
Charlotte G. Mortz-2015	2015	White	Cohort study	Diagnosed	Diagnosed	306	80	111	6	36.27451	Europe	No
Jennifer Whitele-2016	2016	Mixed	Cross-sectional study	Self-report	Self-report	428	148	96	6	22.42991	North America	No
Y.M.F. Andersen-2017	2017	White	Cohort study	Diagnosed	Diagnosed	7937		1430	8	18.01688	Europe	Unclear
Saisindhu Narala-2017	2017	White	Cross-sectional study	Self-report	Self-report	2607		651	6	24.97123	North America	Unclear
J.S. Lee-2017	2017	Asian	Cross-sectional study	Diagnosed	Diagnosed	1325		87	6	6.566038	Asia	Unclear
H. Yemaneberhan-2004	2004	Mixed	Cross-sectional study	Self-report	Self-report	153		68	6	44.44444	Africa	Unclear
Mari Helen Sandström Falk-2006	2006	White	Cohort study	Diagnosed	Diagnosed	42		21	7	50	Europe	Yes
EK Greisenegger-2010	2010	White	Case-control	Diagnosed	Self-report	462		130	5	28.13853	Europe	Unclear
K. Reginald-2010	2010	White	Case-control	Diagnosed	Diagnosed	79		33	5	41.77215	Europe	Yes
Hermenio Lima-2024	2024	White	Cohort study	Diagnosed	Diagnosed	267	153	98	7	36.70412	Global	Yes
April W Armstrong-2024	2024	White	Cohort study	Diagnosed	Diagnosed	100	45	12	4	12	North America	Yes

Author+Year	Publication Year	Ethnity	Design	AD-Definition	Asthma-Definition	Total number in AD	Total male number in AD	Total asthma number in AD	NOS	Rate (%)	Region	Severity
Hilde Lapeere - 2024	2024	Asian	Cohort study	Diagnosed	Diagnosed	118	84	27	5	22.88136	Asia	Yes
Katrina Abuabara-2018	2019	White	Cross-sectional study	Diagnosed	Diagnosed	985202		290828	6	29.51963	Europe	Unclear
Achten, R-2021	2021	White	Cohort study	Diagnosed	Diagnosed	33	16	23	5	69.69697	Europe	Yes
Alexis, A.-2025	2025	White	Clinical trial	Diagnosed	Diagnosed	727	397	231	/	31.77442	Global	Yes
Ali, Z.-2023	2023	White	Cross-sectional study	Self-report	Self-report	53	5	40	6	75.4717	Europe	Yes
Ali, Z.-2022	2022	White	Cohort study	Diagnosed	Diagnosed	12409		819	7	6.600048	Europe	Unclear
Allali, N.-2023	2023	White	Cohort study	Diagnosed	Diagnosed	3216	1698	925	4	28.76244	Europe	Yes
Armario-Hita, J. C.-2024	2024	White	Clinical trial	Diagnosed	Diagnosed	76	34	26	/	34.21053	Europe	Yes
Armstrong, A. W.-2024	2024	Mixed	Clinical trial	Diagnosed	Diagnosed	100	45	12	/	12	North America	Yes
Arruda, L. K.-2021	2021	Mixed	Cohort study	Diagnosed	Diagnosed	187	82	150	4	80.2139	South America	Yes
Bagel, J.-2022	2022	White	Cohort study	Diagnosed	Diagnosed	315	141	203	5	64.44444	North America	Yes
Blauvelt, A.-2017	2017	White	Clinical trial	Diagnosed	Diagnosed	740	446	291	/	39.32432	Global	Yes
Bouazzi, D.-2021	2021	White	Cross-sectional study	Diagnosed	Diagnosed	142		56	/	39.43662	Europe	No
Calzavara-Pinton, P.-2023	2023	Mixed	Cohort study	Diagnosed	Diagnosed	952		327	5	34.34874	Global	Yes
Campanati, A.-2022	2022	White	Case-control	Diagnosed	Diagnosed	684	356	233	7	34.06433	Europe	Yes
Cork, M. J.-2020	2020	White	Clinical trial	Diagnosed	Diagnosed	76	35	30	/	39.47368	Europe	Yes
Darbellay, B.-2024	2024	White	Cross-sectional study	Diagnosed	Diagnosed	94	48	25	5	26.59574	Europe	Yes
de Bruin-Weller, M.-2018	2018	White	Clinical trial	Diagnosed	Diagnosed	325	199	135	/	41.53846	Europe	Yes

Author+Year	Publication Year	Ethnity	Design	AD-Definition	Asthma-Definition	Total number in AD	Total male number in AD	Total asthma number in AD	NOS	Rate (%)	Region	Severity
Deleuran, M.-2020	2020	White	Clinical trial	Diagnosed	Diagnosed	1491	894	637	/	42.723	Global	Yes
Gargiulo, L.-2025	2025	White	Case-control	Diagnosed	Diagnosed	3519	1867	997	8	28.33191	Europe	Yes
Gargiulo, L.-2024	2024	Mixed	Case-control	Diagnosed	Diagnosed	31	20	4	6	12.90323	Global	Yes
Gargiulo, L.-2023	2023	Mixed	Cohort study	Diagnosed	Diagnosed	40	21	4	6	10	Global	Yes
Gooderham, M.-2024	2024	Asian	Clinical trial	Diagnosed	Diagnosed	267	158	68	/	25.46816	Global	Yes
Gooderham, M. J.-2019	2019	White	Clinical trial	Diagnosed	Diagnosed	267	95	1	/	0.374532	Global	Yes
Gutermuth, J.-2022	2022	White	Clinical trial	Diagnosed	Diagnosed	277	165	12	/	4.33213	Europe	Yes
Lee, D. H.-2022	2022	Asian	Case-control	Diagnosed	Diagnosed	27	20	3	5	11.11111	Asia	Yes
Lee, H.-2022	2022	Asian	Case-control	Diagnosed	Diagnosed	40	29	12	5	30	Asia	Yes
Londoño, J.-2021	2021	White	Cohort study	Diagnosed	Diagnosed	93	56	39	5	41.93548	South America	Yes
Melgosa Ramos, F. J.-2026	2026	Mixed	Cohort study	Diagnosed	Diagnosed	38	16	17	7	44.73684	Europe	Yes
Mora, T.-2024	2024	White	Cohort study	Diagnosed	Diagnosed	537098		46190	5	8.59992	Europe	Yes
Napolitano, M.-2020	2020	White	Cohort study	Diagnosed	Diagnosed	30	17	2	7	6.666667	Europe	Yes
Napolitano, M.-2023	2023	White	Cohort study	Diagnosed	Diagnosed	16	8	3	6	18.75	Europe	Yes
Nettis, E.-2020	2020	White	Cohort study	Diagnosed	Diagnosed	123	72	58	7	47.15447	Europe	Yes
Quint, T.-2020	2020	White	Cohort study	Diagnosed	Diagnosed	94	59	26	6	27.65957	Europe	Yes
Saeki, H.-2023	2023	Asian	Cohort study	Diagnosed	Diagnosed	599	414	2	6	0.33389	Asia	Yes
Schmid-Grendelmeier, P.-2024	2024	Mixed	Clinical trial	Diagnosed	Diagnosed	942		166	/	17.62208	Global	Yes
Sher, E.-2023	2023	Mixed	Clinical trial	Diagnosed	Diagnosed	851		261	/	30.6698	Global	Yes
Simpson, E. L.-	2022	White	Clinical	Diagnosed	Diagnosed	1596	947	616	/	38.5964	Global	Yes

Author+Year	Publication Year	Ethnity	Design	AD-Definition	Asthma-Definition	Total number in AD	Total male number in AD	Total asthma number in AD	NOS	Rate (%)	Region	Severity
2022-1			trial							9		
Simpson, E. L.-2022-2	2022	White	Clinical trial	Diagnosed	Diagnosed	380	209	177	/	46.57895	Global	Yes
Thyssen, J. P.-2023-1	2023	White	Clinical trial	Diagnosed	Diagnosed	972	629	441	/	45.37037	Global	Yes
Thyssen, J. P.-2023-2	2023	White	Clinical trial	Diagnosed	Diagnosed	483	298	230	/	47.61905	Global	Yes
Thyssen, J. P.-2023-3	2023	White	Clinical trial	Diagnosed	Diagnosed	521	369	270	/	51.82342	Global	Yes
Thyssen, J. P.-2023-4	2023	White	Clinical trial	Diagnosed	Diagnosed	264	178	138	/	52.27273	Global	Yes
Orfali RL-2013	2013	White	Cross-sectional study	Diagnosed	Diagnosed	80		54	5	67.5	Global	Unclear
Pesce G-2015	2015	White	Cross-sectional study	Diagnosed+ Self-report	Diagnosed+ Self-report	819		347	7	42.36874	Europe	Unclear
Simpson EL-2016	2016	White	Clinical trial	Diagnosed	Diagnosed	380		153	/	40.26316	Global	Yes
Silverberg JI-2018	2018	White	Cohort study	Diagnosed	Diagnosed	356		130	7	36.51685	North America	Unclear
Sicras Mainar A-2017	2017	White	Case-control	Diagnosed	Diagnosed	6186		1027	9	16.602	Europe	No
I. Annesi-Maesano-2006	2006	White	Cross-sectional study	Diagnosed	Diagnosed	458		110	7	24.01747	Europe	Unclear
Johanna M. Mandelin-2010	2010	White	Cohort study	Diagnosed	Diagnosed	50		10	8	20	Europe	Unclear

Table S3. Table of Literature characteristics on the association between asthma prevalence and atopic dermatitis

Author +Year	Publication Year	Ethnity	Design	AD-Definition	Asthma-Definition	Total number in AD	Total asthma number in AD	Total number in control	Total asthma number in control	Type of control	NOS	OR	Region	Severity
Katrina Abuabara-2019-1	2019	White	Cohort study	Diagnosed	Diagnosed	2318	807	10825	2362	1	7	1.91361	Europe	Unclear
Katrina Abuabara-2019-2	2019	White	Cohort study	Diagnosed	Diagnosed	3275	1005	8611	1451	1	7	2.18467	Europe	Unclear
Ali, Z.-2021	2021	White	Cross-sectional study	Diagnosed	Diagnosed	8095	1201	32380	878	1	6	6.250508	Europe	Unclear
Alqahtani, J. M.-2020	2020	White	Cross-sectional study	Self-report	Self-report	10	5	222	60	2	6	2.7	Asia	Unclear
Arima, K.-2018	2018	Asian	Cross-sectional study	Diagnosed	Diagnosed	634	80	1268	31	1	9	5.762199	Asia	Yes
Ito, Y.-2023	2023	Asian	Cross-sectional study	Diagnosed +self-report	Diagnosed +self-report	2910	966	11626	1887	1	6	2.564622	Asia	Yes
Mao, D.-2023	2023	Asian	Cross-sectional study	Diagnosed	Diagnosed	2202	59	34498	369	1	10	2.546402	Asia	Yes
Smirnova, J.-2020	2020	White	Cross-sectional study	Diagnosed	Diagnosed	4175	570	25955	1691	1	8	2.268759	Europe	Yes
Ben-Shoshan M-2015	2015	White	Case-control	Diagnosed +self-report	Diagnosed	184	161	4950	549	1	7	56.11475	North America	Unclear
Moshe S-2015-1	2015	White	Cohort study	Diagnosed	Diagnosed	89	1	26921	575	1	6	0.520672	Asia	Unclear
Moshe S-2015-2	2015	White	Cohort study	Diagnosed	Diagnosed	70	3	38488	661	1	6	2.5624	Asia	Unclear
Moshe S-2015-	2015	White	Cohort study	Diagnosed	Diagnosed	231	5	123623	1848	1	6	1.45786	Asia	Unclear

Author +Year	Publication Year	Ethnity	Design	AD-Definition	Asthma-Definition	Total number in AD	Total asthma number in AD	Total number in control	Total asthma number in control	Type of control	NOS	OR	Region	Severity
3												6		
Luukkonen TM-2017	2017	White	Cohort study	Diagnosed	Diagnosed	445	202	1664	137	1	8	9.265387	Europe	Unclear
Morgan BW-2018	2018	Black	Cross-sectional study	Diagnosed	Diagnosed	109	18	1199	89	1	7	2.466971	Africa	Unclear
Radtke MA-2017	2017	White	Cross-sectional study	Diagnosed	Diagnosed	48140	8553	1264075	72052	1	6	3.574411	Europe	Unclear
Eriksson UK-2008	2008	White	Cohort study	Self-report	Self-report	303	3	7342	98	1	9	0.739184	Europe	Unclear
Liborija Lugovic-1997	1997	White	Cohort study	Diagnosed	Diagnosed	80	9	81	2	1	6	5.007042	Europe	Yes
H. Kyllönen-2006	2006	White	Cross-sectional study	Diagnosed	Diagnosed	86	31	49	1	1	5	27.05455	Europe	Yes
Kerstin Bingefors-2013	2013	White	Cross-sectional study	Self-report	Self-report	666	78	4209	257	2	7	2.039863	Europe	Unclear
Jonathan I. Silverberg-2013	2013	Mixed	Cross-sectional study	Self-report	Diagnosed	2641	570	24498	2779	2	7	2.151028	North America	Unclear
Charlotte G. Mortz-2015	2015	White	Cohort study	Diagnosed	Diagnosed	306	111	593	70	1	6	4.252967	Europe	No
Jennifer Whitele-2016	2016	Mixed	Cross-sectional study	Self-report	Self-report	428	96	74572	5891	2	6	3.371171	North America	No
Y.M.F. Andersen-2017	2017	White	Cohort study	Diagnosed	Diagnosed	7937	1430	79370	1567	2	8	10.91145	Europe	Unclear
Saisindhu	2017	White	Cross-sectional	Self-report	Self-report	2607	651	24208	2800	2	6	2.54466	North America	Unclear

Author +Year	Publication Year	Ethnity	Design	AD-Definition	Asthma-Definition	Total number in AD	Total asthma number in AD	Total number in control	Total asthma number in control	Type of control	NOS	OR	Region	Severity
Narala-2017			study									3		
J.S. Lee-2017	2017	Asian	Cross-sectional study	Diagnosed	Diagnosed	1325	87	46026	1393	2	6	2.25 166 4	Asia	Unclear
H. Yemane berhan-2004	2004	Mixed	Cross-sectional study	Self-report	Self-report	153	68	12723	338	1	6	29.3 136 1	Africa	Unclear
Mari Helen Sandstr öm Falk-2006	2006	White	Cohort study	Diagnosed	Diagnosed	42	21	37	11	2	7	2.36 363 6	Europe	Yes
K. Reginal d-2010	2010	White	Case-control	Diagnosed	Diagnosed	79	33	87	37	1	5	0.96 944 8	Europe	Yes

Table S4. NOS score of literature

Author+Year	Publication Year	Design	selection of study population	comparability	ascertainment of exposure/outcome	Total
Katrina Abuabara-2019	2019	Cohort study	4	2	1	7
Katrina Abuabara-2019	2019	Cohort study	4	2	1	7
Ali, Z.-2021	2021	Cross-sectional study	3	1	2	6
Alqahtani, J. M.-2020	2020	Cross-sectional study	3	2	1	6
Arima, K.-2018	2018	Cross-sectional study	4	2	3	9
Ito, Y.-2023	2023	Cross-sectional study	2	1	3	6
Mao, D.-2023	2023	Cross-sectional study	5	2	3	10

Author+Year	Publication Year	Design	selection of study population	comparability	ascertainment of exposure/outcome	Total
Smirnova, J.-2020	2020	Cross-sectional study	3	2	3	8
Ben-Shoshan M-2015	2015	Case-report	3	2	2	7
Moshe S-2015	2015	Cohort study	3	1	2	6
Moshe S-2015	2015	Cohort study	3	1	2	6
Moshe S-2015	2015	Cohort study	3	1	2	6
Tanei R-2015	2015	Case-report	3	1	2	6
Luukkonen TM-2017	2017	Cohort study	4	1	3	8
Morgan BW-2018	2018	Cross-sectional study	3	1	3	7
Radtke MA-2017	2017	Cross-sectional study	2	1	3	6
Eriksson UK-2008	2008	Cohort study	4	3	2	9
Liborija Lugovic-1997	1997	Cohort study	3	2	1	6
H. Kyllönen-2006	2006	Cross-sectional study	2	1	2	5
Kerstin Bingefors-2013	2013	Cross-sectional study	3	1	3	7
Jonathan I. Silverberg-2013	2013	Cross-sectional study	3	2	2	7
Charlotte G. Mortz-2015	2015	Cohort study	3	1	2	6
Jennifer Whiteley-2016	2016	Cross-sectional study	3	1	2	6
Y.M.F. Andersen-2017	2017	Cohort study	4	2	2	8
Saisindhu Narala-2017	2017	Cross-sectional study	2	2	2	6
J.S. Lee-2017	2017	Cross-sectional study	3	1	2	6
Peter Montnemery-2003	2003	Cross-sectional study	3	1	2	6
H. Yemaneberhan-2004	2004	Cross-sectional study	2	2	2	6
Mari Helen Sandström Falk-	2006	Cohort study	4	1	2	7

Author+Year	Publication Year	Design	selection of study population	comparability	ascertainment of exposure/outcome	Total
2006						
EK Greisenegger-2010	2010	Case-report	2	1	2	5
K. Reginald-2010	2010	Case-report	2	1	2	5
Hermenio Lima-2024	2024	Cohort study	4	1	2	7
April W Armstrong-2024	2024	Cohort study	3	0	1	4
Hilde Lapeere -2024	2024	Cohort study	3	0	2	5
Katrina Abuabara-2018	2019	Cross-sectional study	3	1	2	6
Achten, R-2021	2021	Cohort study	2	0	3	5
Ali, Z.-2023	2023	Cross-sectional study	2	1	3	6
Ali, Z.-2022	2022	Cohort study	3	1	3	7
Allali, N.-2023	2023	Cohort study	3	0	1	4
Arruda, L. K.-2021	2021	Cohort study	1	2	1	4
Bagel, J.-2022	2022	Cohort study	3	1	1	5
Calzavara-Pinton, P.-2023	2023	Cohort study	3	1	1	5
Campanati, A.-2022	2022	Case-report	3	1	3	7
Darbellay, B.-2024	2024	Cross-sectional study	2	1	2	5
Gargiulo, L.-2025	2025	Case-report	4	1	3	8
Gargiulo, L.-2024	2024	Case-report	2	1	3	6
Gargiulo, L.-2023	2023	Cohort study	2	1	3	6
Lee, D. H.-2022	2022	Case-report	2	1	2	5
Lee, H.-2022	2022	Case-report	2	1	3	5
Londoño, J.-2021	2021	Cohort study	2	1	3	5
Melgosa Ramos, F. J.-2026	2026	Cohort study	3	1	3	7
Mora, T.-2024	2024	Cohort study	3	1	1	5
Napolitano, M.-2020	2020	Cohort study	3	1	3	7
Napolitano, M.-2023	2023	Cohort study	2	1	3	6
Nettis, E.-2020	2020	Cohort study	3	1	3	7
Quint, T.-2020	2020	Cohort study	3	1	2	6
Saeki, H.-2023	2023	Cohort study	3	1	2	6

Author+Year	Publication Year	Design	selection of study population	comparability	ascertainment of exposure/outcome	Total
Orfali RL-2013	2013	Cross-sectional study	2	1	2	5
Pesce G-2015	2015	Cross-sectional study	3	2	2	7
Silverberg JI-2018	2018	Cohort study	3	2	2	7
Sicras Mainar A-2017	2017	Case-report	4	2	3	9
Vincent Yi-Fong Su-2014	2014	Cohort study	4	2	3	9
Päivikki Susitaival-2003	2003	Cross-sectional study	3	2	3	8
I. Annesi-Maesano-2006	2006	Cross-sectional study	2	2	3	7
Johanna M. Mandelin-2010	2010	Cohort study	4	1	3	8
Cabrera, A.-2022	2022	Cross-sectional study	2	2	3	7
Shifti, D. M.-2025	2025	Cross-sectional study	3	1	3	7

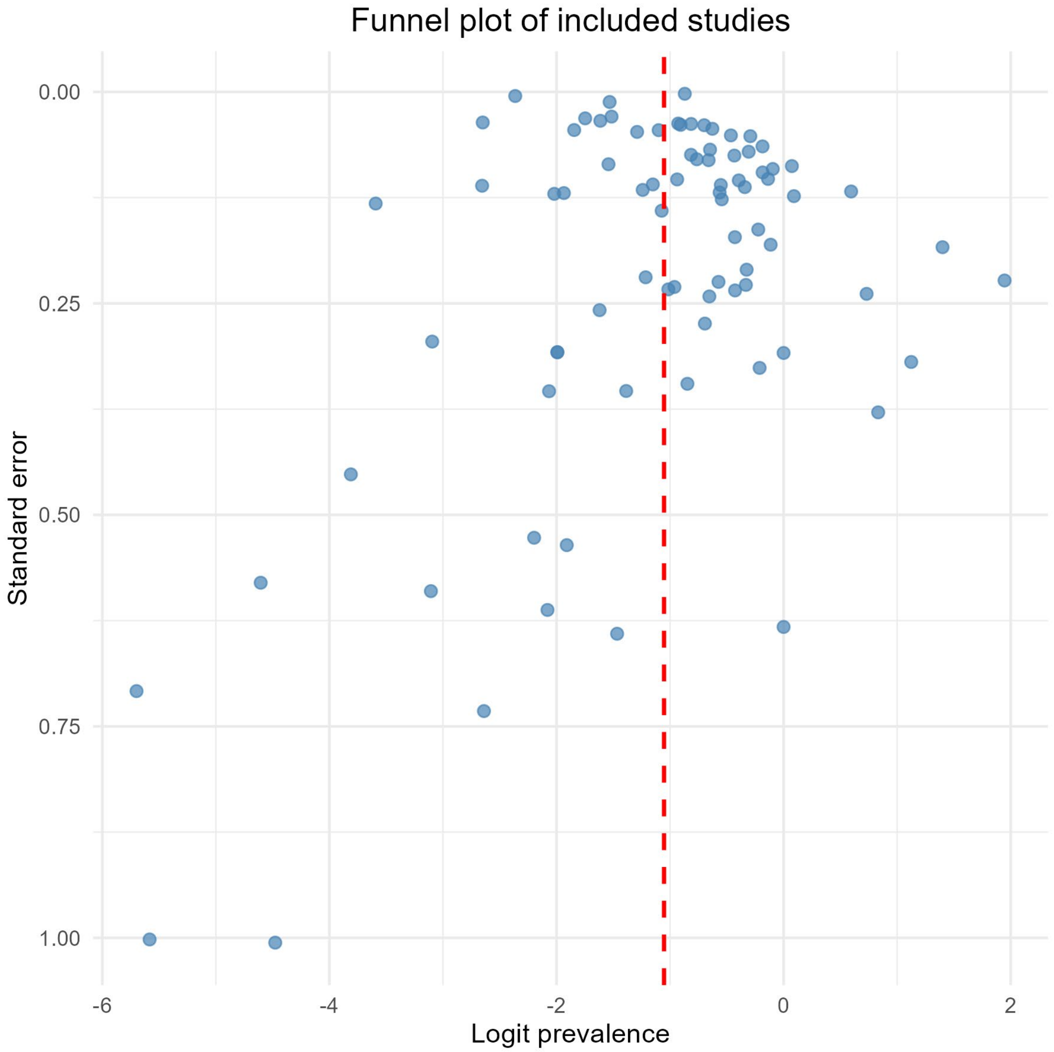

Figure S1. Funnel plot of asthma prevalence

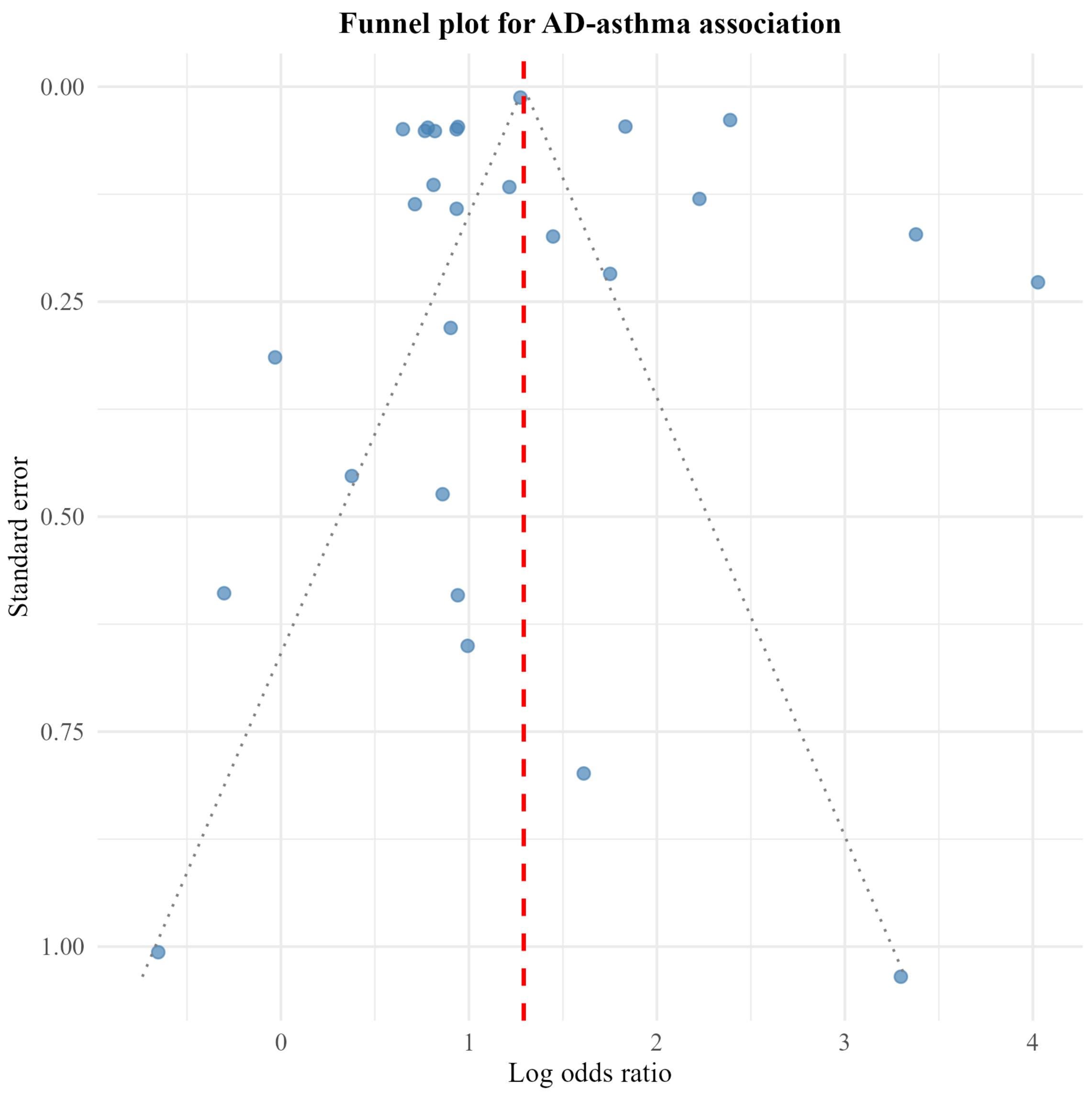

Figure S2. Funnel plot of association between asthma and atopic dermatitis

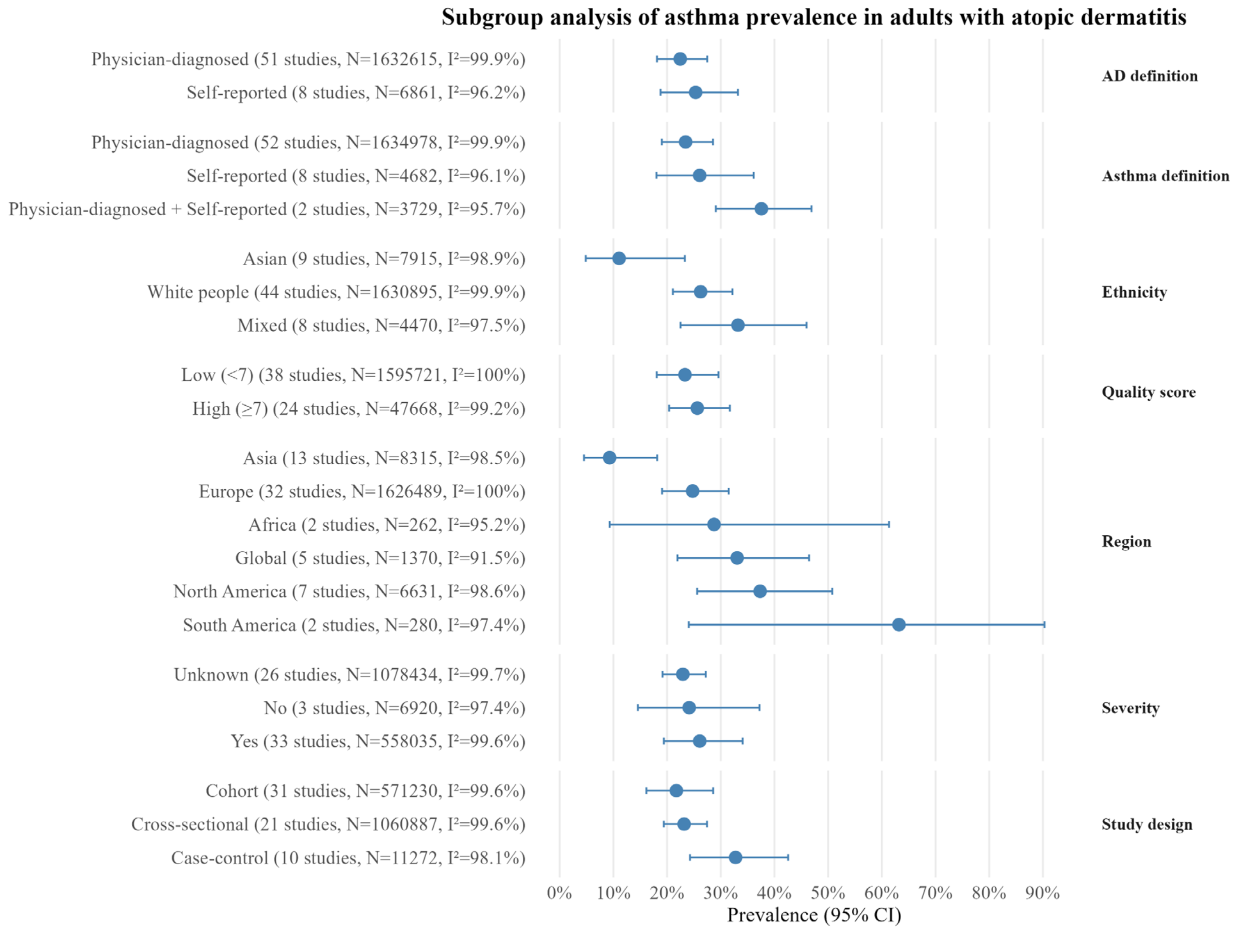

Figure S3. Subgroup analysis of asthma prevalence

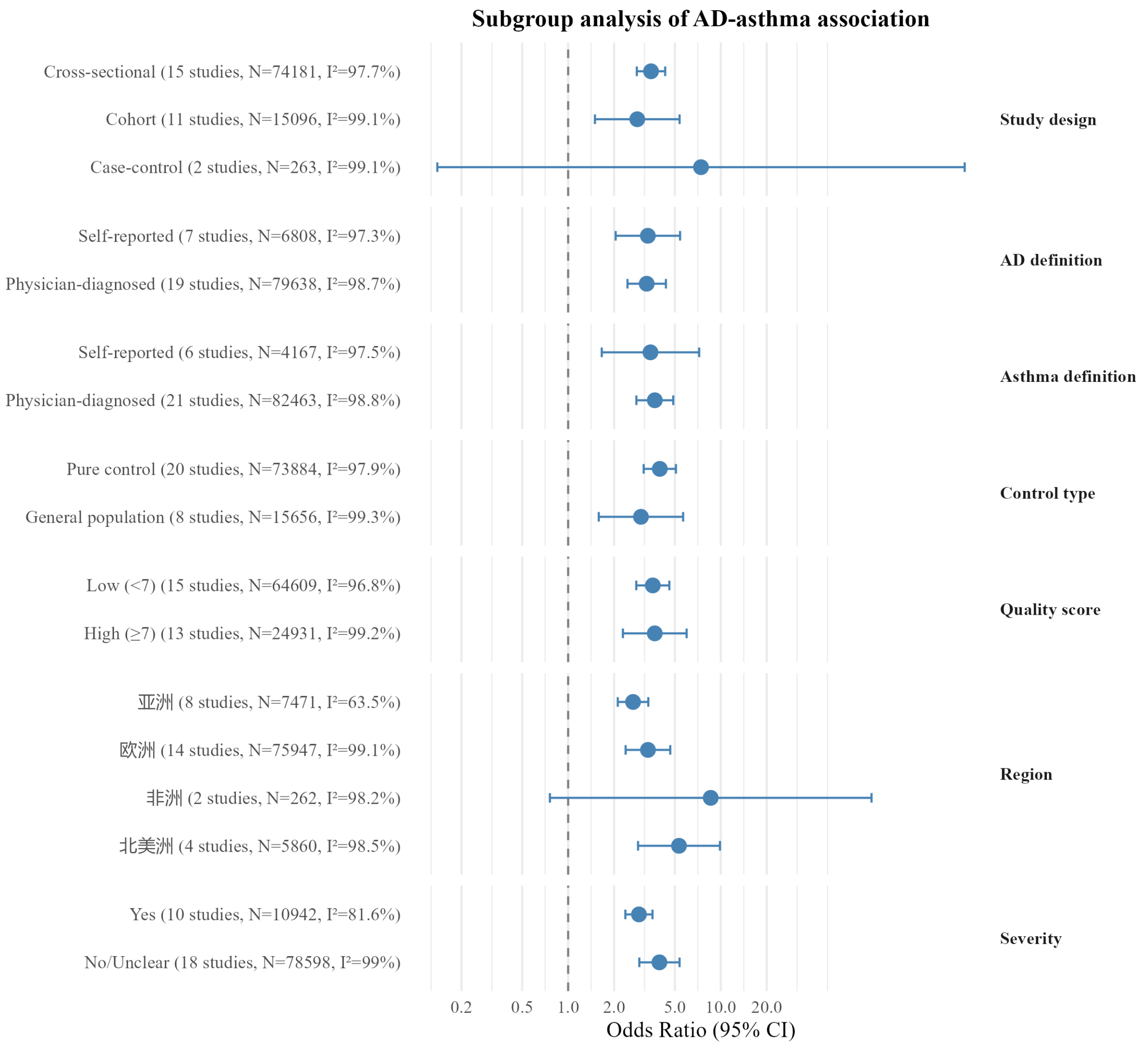

Figure S4. Subgroup analysis of AD -asthma association

Table S5. Sensitivity analysis of asthma prevalence

n_studies	p_combined	ci_lower	ci_upper	tau2	Q	df	I2	removed_study
82	0.25866	0.22111	0.300129	0.835988	85917.8	81	99.90572	None (All studies)
81	0.257487	0.219709	0.29927	0.841632	85781.49	80	99.90674	Katrina Abuabara-2019-1
81	0.257926	0.220044	0.299822	0.844547	85845.86	80	99.90681	Katrina Abuabara-2019-2
81	0.260306	0.222153	0.302463	0.845854	85533.55	80	99.90647	Ali, Z.-2021
81	0.256838	0.219315	0.298328	0.835999	85914.58	80	99.90688	Alqahtani, J. M.-2020
81	0.260891	0.22287	0.302871	0.836483	85873.12	80	99.90684	Arima, K.-2018
81	0.257647	0.219818	0.299487	0.843355	85794.6	80	99.90675	Ito, Y.-2023
81	0.265153	0.226766	0.307455	0.833375	85571.19	80	99.90651	Mao, D.-2023
81	0.260637	0.222566	0.302685	0.840088	85670.15	80	99.90662	Smirnova, J.-2020
81	0.251468	0.214472	0.29247	0.834401	85726.67	80	99.90668	Ben-Shoshan M-2015
81	0.262641	0.224582	0.304615	0.835903	85906.76	80	99.90688	Moshe S-2015-1
81	0.262394	0.224299	0.304421	0.835927	85906.66	80	99.90688	Moshe S-2015-2
81	0.264394	0.226078	0.306632	0.835721	85882.79	80	99.90685	Moshe S-2015-3
81	0.257809	0.220126	0.299466	0.836148	85915.19	80	99.90688	Tanei R-2015
81	0.256448	0.218882	0.298003	0.836477	85818.01	80	99.90678	Luukkonen TM-2017
81	0.260012	0.2221	0.301883	0.836161	85914.27	80	99.90688	Morgan BW-2018
81	0.25865	0.219099	0.302576	0.926836	84782.29	80	99.90564	Radtke MA-2017
81	0.265211	0.226835	0.307496	0.835689	85882.05	80	99.90685	Eriksson UK-2008
81	0.260929	0.22294	0.302867	0.836036	85910.9	80	99.90688	Liborija Lugovic-1997
81	0.257495	0.219837	0.29913	0.836198	85911.53	80	99.90688	H. Kyllonen-2006
81	0.261108	0.223067	0.303105	0.836379	85863.94	80	99.90683	Kerstin Bingefors-2013
81	0.259185	0.221228	0.301136	0.841811	85907.13	80	99.90688	Jonathan I. Silverberg-2013
81	0.257413	0.219743	0.299064	0.836703	85894.63	80	99.90686	Charlotte G. Mortz-2015
81	0.259122	0.221271	0.300944	0.83697	85916.98	80	99.90689	Jennifer Whitele-2016
81	0.259647	0.221479	0.301843	0.850008	85748.25	80	99.9067	Y.M.F. Andersen-2017
81	0.258688	0.220769	0.300606	0.842463	85917.18	80	99.90689	Saisindhu Narala-2017
81	0.262756	0.224571	0.304882	0.835237	85730.04	80	99.90668	J.S. Lee-2017
81	0.256593	0.219022	0.298148	0.836188	85886.34	80	99.90685	H. Yemaneberhan-2004
81	0.256237	0.218723	0.297733	0.836004	85904.26	80	99.90687	Mari Helen Sandstrom Falk-2006

n_studies	p_combined	ci_lower	ci_upper	tau2	Q	df	I2	removed_study
81	0.258349	0.220573	0.300104	0.837193	85914.13	80	99.90688	EK Greisenegger-2010
81	0.256916	0.21932	0.298493	0.83613	85905.39	80	99.90687	K. Reginald-2010
81	0.25737	0.219708	0.299015	0.836603	85896.15	80	99.90686	Hermenio Lima-2024
81	0.260834	0.222848	0.302773	0.836061	85910.05	80	99.90688	April W Armstrong-2024
81	0.25905	0.22123	0.300838	0.836271	85917.67	80	99.90689	Hilde Lapeere -2024
81	0.259835	0.224108	0.299062	0.731139	23699.32	80	99.66244	Katrina Abuabara-2018
81	0.254548	0.217224	0.295862	0.835826	85890.79	80	99.90686	Achten, R-2021
81	0.257896	0.220151	0.299625	0.837863	85896.03	80	99.90686	Alexis, A.-2025
81	0.253709	0.216466	0.294946	0.835639	85867.7	80	99.90683	Ali, Z.-2023
81	0.262893	0.22482	0.304877	0.828838	84159.19	80	99.90494	Ali, Z.-2022
81	0.258164	0.22026	0.30008	0.84441	85883.22	80	99.90685	Allali, N.-2023
81	0.257699	0.220022	0.299351	0.836184	85913.83	80	99.90688	Armario-Hita, J. C.-2024
81	0.260834	0.222848	0.302773	0.836061	85910.05	80	99.90688	Armstrong, A. W.-2024
81	0.252661	0.215533	0.293789	0.834529	85726.99	80	99.90668	Arruda, L. K.-2021
81	0.254526	0.217193	0.295852	0.83484	85701.61	80	99.90665	Bagel, J.-2022
81	0.257058	0.219408	0.298697	0.837476	85830.72	80	99.90679	Blauvelt, A.-2017
81	0.257108	0.219483	0.298714	0.836279	85900.86	80	99.90687	Bouazzi, D.-2021
81	0.25759	0.219868	0.299302	0.838331	85866.65	80	99.90683	Calzavara-Pinton, P.-2023
81	0.257634	0.21992	0.299334	0.837684	85883.07	80	99.90685	Campanati, A.-2022
81	0.257151	0.21953	0.29875	0.836148	85908.69	80	99.90688	Cork, M. J.-2020
81	0.258565	0.220797	0.300303	0.836237	85917.54	80	99.90689	Darbellay, B.-2024
81	0.256853	0.219244	0.298447	0.836552	85868.03	80	99.90683	de Bruin-Weller, M.-2018
81	0.256682	0.219054	0.298306	0.838282	85658.42	80	99.90661	Deleuran, M.-2020
81	0.258207	0.220283	0.300146	0.845192	85886.9	80	99.90685	Gargiulo, L.-2025
81	0.260299	0.222405	0.30214	0.836023	85915.72	80	99.90689	Gargiulo, L.-2024
81	0.260869	0.222916	0.302767	0.836006	85913.75	80	99.90688	Gargiulo, L.-2023
81	0.258702	0.220903	0.300475	0.836663	85917.61	80	99.90689	Gooderham, M.-2024
81	0.263958	0.225768	0.306054	0.835819	85898.09	80	99.90687	Gooderham, M. J.-2019
81	0.263447	0.225201	0.305625	0.83572	85873.74	80	99.90684	Gutermuth, J.-2022
81	0.26048	0.222581	0.302323	0.83601	85915.43	80	99.90689	Lee, D. H.-2022
81	0.258196	0.220484	0.299877	0.836102	85917.1	80	99.90689	Lee, H.-2022
81	0.256884	0.219288	0.298461	0.836151	85902.93	80	99.90687	Londono, J.-2021

n_studies	p_combined	ci_lower	ci_upper	tau2	Q	df	I2	removed_study
81	0.256745	0.21918	0.298287	0.836043	85909.77	80	99.90688	Melgosa Ramos, F. J.-2026
81	0.276878	0.252613	0.30253	0.272994	10325.46	80	99.22522	Mora, T.-2024
81	0.261143	0.223197	0.303025	0.835981	85913.58	80	99.90688	Napolitano, M.-2020
81	0.259371	0.221589	0.301103	0.836027	85917.54	80	99.90689	Napolitano, M.-2023
81	0.25634	0.218799	0.297866	0.836089	85885.8	80	99.90685	Nettis, E.-2020
81	0.258435	0.22068	0.300161	0.83624	85917.23	80	99.90689	Quint, T.-2020
81	0.266171	0.227722	0.308517	0.83562	85876.3	80	99.90684	Saeki, H.-2023
81	0.259887	0.221944	0.3018	0.83758	85895.2	80	99.90686	Schmid-Grendelmeier, P.-2024
81	0.258021	0.220257	0.299771	0.838203	85899.24	80	99.90687	Sher, E.-2023
81	0.257104	0.219412	0.298794	0.839328	85747.04	80	99.9067	Simpson, E. L.-2022-1
81	0.256334	0.218783	0.297873	0.836323	85823.46	80	99.90679	Simpson, E. L.-2022-2
81	0.25643	0.218852	0.297998	0.83705	85700.17	80	99.90665	Thyssen, J. P.-2023-1
81	0.256222	0.218682	0.297751	0.836312	85787.33	80	99.90675	Thyssen, J. P.-2023-2
81	0.2558	0.218313	0.297277	0.835856	85727.67	80	99.90668	Thyssen, J. P.-2023-3
81	0.25578	0.218296	0.297253	0.835898	85818.66	80	99.90678	Thyssen, J. P.-2023-4
81	0.254393	0.217069	0.295711	0.835633	85856.65	80	99.90682	Orfali RL-2013
81	0.256739	0.219125	0.298342	0.837295	85780.57	80	99.90674	Pesce G-2015
81	0.25698	0.219354	0.298591	0.836712	85867.57	80	99.90683	Simpson EL-2016
81	0.257382	0.219713	0.299033	0.836811	85889.77	80	99.90686	Silverberg JI-2018
81	0.259967	0.221857	0.302082	0.845467	85721.18	80	99.90667	Sicras Mainar A-2017
81	0.258894	0.221064	0.300699	0.837098	85917.78	80	99.90689	I. Annesi-Maesano-2006
81	0.259404	0.22157	0.301199	0.836098	85917.3	80	99.90689	Johanna M. Mandelin-2010

Table S6. Sensitivity analysis of AD -asthma association

n_studies	OR	ci_low	ci_high	tau2	Q	df	I2	removed_study
28	3.638533	2.893907	4.574757	0.300774	1946.771	27	98.61309	None (All studies)
27	3.750088	2.96594	4.741553	0.301602	1782.685	26	98.54153	Katrina Abuabara-2019-1
27	3.722463	2.932706	4.724897	0.313228	1836.929	26	98.58459	Katrina Abuabara-2019-2
27	3.544989	2.798472	4.490645	0.307191	1792.271	26	98.54933	Ali, Z.-2021
27	3.659375	2.903838	4.611492	0.301057	1946.59	26	98.66433	Alqahtani, J. M.-2020
27	3.570666	2.82609	4.511411	0.301545	1941.861	26	98.66108	Arima, K.-2018
27	3.690656	2.896889	4.70192	0.324567	1895.297	26	98.62818	Ito, Y.-2023

n_studies	OR	ci_low	ci_high	tau2	Q	df	I2	removed_study
27	3.69577	2.922175	4.674159	0.303322	1941.182	26	98.66061	Mao, D.-2023
27	3.715571	2.926783	4.716943	0.31376	1867.831	26	98.60801	Smirnova, J.-2020
27	3.266232	2.605429	4.094632	0.279028	1799.491	26	98.55515	Ben-Shoshan M-2015
27	3.713604	2.950424	4.674195	0.300424	1943.124	26	98.66195	Moshe S-2015-1
27	3.665887	2.908267	4.620872	0.301069	1946.462	26	98.66424	Moshe S-2015-2
27	3.732039	2.959066	4.70693	0.30064	1942.883	26	98.66178	Moshe S-2015-3
27	3.490376	2.766252	4.404055	0.296233	1892.683	26	98.62629	Luukkonen TM-2017
27	3.691434	2.922869	4.662093	0.301496	1945.061	26	98.66328	Morgan BW-2018
27	3.586767	2.658194	4.839715	0.526907	1946.472	26	98.66425	Radtke MA-2017
27	3.765628	2.98844	4.744934	0.300003	1939.65	26	98.65955	Eriksson UK-2008
27	3.621352	2.875175	4.561179	0.301005	1946.588	26	98.66433	Liborija Lugovic-1997
27	3.566109	2.833418	4.488267	0.30039	1942.93	26	98.66182	H. Kyllonen-2006
27	3.733345	2.9533	4.719421	0.301826	1930.085	26	98.65291	Kerstin Bingefors-2013
27	3.726081	2.938259	4.725136	0.310463	1847.219	26	98.59248	Jonathan I. Silverberg-2013
27	3.613498	2.85795	4.568788	0.302936	1945.722	26	98.66374	Charlotte G. Mortz-2015
27	3.64947	2.882894	4.619884	0.305767	1946.553	26	98.66431	Jennifer Whitele-2016
27	3.485332	2.877799	4.221122	0.187622	1055.525	26	97.53677	Y.M.F. Andersen-2017
27	3.693012	2.901677	4.700159	0.32146	1898.975	26	98.63084	Saisindhu Narala-2017
27	3.717822	2.939152	4.702785	0.303438	1930.579	26	98.65325	J.S. Lee-2017
27	3.331504	2.656365	4.178237	0.279306	1795.623	26	98.55203	H. Yemaneberhan-2004
27	3.680539	2.918123	4.642149	0.301105	1946.025	26	98.66394	Mari Helen Sandstrom Falk-2006
27	3.813147	3.022559	4.810523	0.298909	1929.681	26	98.65263	K. Reginald-2010

Table S7. List of studies included in the meta-analysis (corresponding to Study 1–28 in Figure 2).

Study_Number	Reference
Study 1	Katrina Abuabara-2019-1
Study 2	Katrina Abuabara-2019-2
Study 3	Ali, Z.-2021
Study 4	Alqahtani, J. M.-2020
Study 5	Arima, K.-2018
Study 6	Ito, Y.-2023
Study 7	Mao, D.-2023

Study 8	Smirnova, J.-2020
Study 9	Ben-Shoshan M-2015
Study 10	Moshe S-2015-1
Study 11	Moshe S-2015-2
Study 12	Moshe S-2015-3
Study 13	Luukkonen TM-2017
Study 14	Morgan BW-2018
Study 15	Radtke MA-2017
Study 16	Eriksson UK-2008
Study 17	Liborija Lugovic-1997
Study 18	H. Kyllönen-2006
Study 19	Kerstin Bingefors-2013
Study 20	Jonathan I. Silverberg-2013
Study 21	Charlotte G. Mortz-2015
Study 22	Jennifer Whitele-2016
Study 23	Y.M.F. Andersen-2017
Study 24	Saisindhu Narala-2017
Study 25	J.S. Lee-2017
Study 26	H. Yemaneberhan-2004
Study 27	Mari Helen Sandström Falk-2006
Study 28	K. Reginald-2010
